# Preliminary Estimates of Effectiveness of Monovalent mRNA Vaccines in Preventing Symptomatic SARS-CoV-2 Infection Among Children Aged 3–5 Years — Increasing Community Access to Testing Program, United States, July 2022–February 2023

**DOI:** 10.15585/mmwr.mm7207a3

**Published:** 2023-02-17

**Authors:** Katherine E. Fleming-Dutra, Allison Avrich Ciesla, Lauren E. Roper, Zachary R. Smith, Joseph D. Miller, Emma K. Accorsi, Jennifer R. Verani, Nong Shang, Gordana Derado, Ryan E. Wiegand, Tamara Pilishvili, Amadea Britton, Ruth Link-Gelles

**Affiliations:** ^1^National Center for Immunization and Respiratory Diseases, CDC; ^2^Eagle Health Analytics, San Antonio, Texas; ^3^Center for Preparedness and Response, CDC; ^4^Epidemic Intelligence Service, CDC.

On June 18, 2022, the Advisory Committee on Immunization Practices (ACIP) issued interim recommendations for use of the 2-dose monovalent Moderna COVID-19 vaccine as a primary series for children aged 6 months–5 years* and the 3-dose monovalent Pfizer-BioNTech COVID-19 vaccine as a primary series for children aged 6 months–4 years,^†^ based on safety, immunobridging, and limited efficacy data from clinical trials (*1*–*3*). Monovalent mRNA vaccine effectiveness (VE) against symptomatic SARS-CoV-2 infection was evaluated using the Increasing Community Access to Testing (ICATT) program, which provides SARS-CoV-2 testing to persons aged ≥3 years at pharmacy and community-based testing sites nationwide^§^ (*4*,*5*). Among children aged 3–5 years with one or more COVID-19–like illness symptoms^¶^ for whom a nucleic acid amplification test (NAAT) was performed during August 1, 2022–February 5, 2023, VE of 2 monovalent Moderna doses (complete primary series) against symptomatic infection was 60% (95% CI = 49% to 68%) 2 weeks–2 months after receipt of the second dose and 36% (95% CI = 15% to 52%) 3–4 months after receipt of the second dose. Among symptomatic children aged 3–4 years with NAATs performed during September 19, 2022–February 5, 2023, VE of 3 monovalent Pfizer-BioNTech doses (complete primary series) against symptomatic infection was 31% (95% CI = 7% to 49%) 2 weeks–4 months after receipt of the third dose; statistical power was not sufficient to estimate VE stratified by time since receipt of the third dose. Complete monovalent Moderna and Pfizer-BioNTech primary series vaccination provides protection for children aged 3–5 and 3–4 years, respectively, against symptomatic infection for at least the first 4 months after vaccination. CDC expanded recommendations for use of updated bivalent vaccines to children aged ≥6 months on December 9, 2022 (*6*), which might provide increased protection against currently circulating SARS-CoV-2 variants (*7*,*8*). Children should stay up to date with recommended COVID-19 vaccines, including completing the primary series; those who are eligible should receive a bivalent vaccine dose.

ICATT is a CDC program** that contracts with pharmacy- and community-based testing vendors to provide no-cost SARS-CoV-2 testing nationwide (*4*,*5*). At registration, caregivers of minors report information on the presence of COVID-19–like illness symptoms, previous SARS-CoV-2 infection,^††^ underlying health conditions,^§§^ and COVID-19 vaccination status. Caregivers are asked to report total number of COVID-19 vaccine doses received, the manufacturer of each dose, and the month and year of receipt of the most recent dose.^¶¶^ Testing vendors report SARS-CoV-2 test data directly to CDC, including collection date and result.

NAATs from children with one or more COVID-19–like illness symptom were eligible for inclusion in the test-negative design case-control study. Tests from children were excluded if the caregiver reported any of the following conditions: immunocompromise, positive SARS-CoV-2 test within 3 months, receipt of a non-mRNA COVID-19 vaccine or mixed product regimen,*** COVID-19 vaccine dose receipt within 2 weeks of test date,^†††^ or third COVID-19 vaccine dose received during or after December 2022 (when bivalent vaccines were recommended for this age group).^§§§^ Data included NAATs performed among children aged 3–5 years (Moderna analysis) and aged 3–4 years (Pfizer-BioNTech analysis).^¶¶¶^ VE, stratified by vaccine product and dose number, was estimated by comparing odds of COVID-19 vaccination versus being unvaccinated in case-patients (those who received a positive SARS-CoV-2 test result) and control-patients (those who received a negative test result). VE was calculated as (1 − adjusted odds ratio) x 100.**** Analysis periods varied for each product and dose combination. Children became eligible to be included in each analysis 2 weeks after the initial date a child could have received each product and dose combination, affecting comparability of product-specific estimates.^††††^ VE for a partial series (1 dose of Moderna; 1 or 2 doses of Pfizer-BioNTech) was assessed from 2 weeks after receipt of the most recent dose through the recommended interval to the next dose.^§§§§^ Consistent with previous studies (*7*,*8*), VE estimates with 95% CI width >50 percentage points were considered imprecise and not reported. This activity was reviewed by CDC and was conducted consistent with applicable federal law and CDC policy.^¶¶¶¶^

Among NAATs performed through ICATT during July 4–February 5, 2023, among children with one or more COVID-19–like illness symptom (before applying exclusion criteria), 18%, 17%, and 26% of those aged 3, 4, and 5 years, respectively, had received ≥1 COVID-19 vaccine dose.***** After applying exclusion criteria, 37,010 NAATs performed at 8,741 ICATT testing sites for children aged 3–5 years were included in the Moderna VE analysis, and 24,094 NAATs performed at 7,615 ICATT testing sites for children aged 3–4 years were included in the Pfizer-BioNTech VE analysis. In the Moderna analysis, 26%, 39% and 35% of children were aged 3, 4, and 5 years, respectively; in the Pfizer-BioNTech analysis, 40% and 60% of children were aged 3 and 4 years, respectively (Table 1). VE of one monovalent Moderna dose (partial primary series) was 40% at 2 weeks–1 month after dose 1 (Table 2). VE of two monovalent Moderna doses (complete primary series) was 60% at 2 weeks–2 months after dose 2 and 36% at 3–4 months. VE of one monovalent Pfizer-BioNTech dose (partial primary series) was 19% at 2 weeks–1 month after dose 1. VE of 2 monovalent Pfizer-BioNTech doses (partial primary series) was 40% at 2 weeks–3 months after dose 2, reflecting the interval between doses 2 and 3. VE of three monovalent Pfizer-BioNTech doses (complete primary series) was 31% at 2 weeks–4 months after dose 3; statistical power was not sufficient to estimate VE stratified by time since dose 3.

**TABLE 1:** Characteristics of children aged 3–5 years with symptoms of COVID-19–like illness and SARS-CoV-2 nucleic acid amplification test results — Increasing Community Access to Testing program, United States, July 4, 2022–February 5, 2023

Characteristic	No. (column %)			
	Moderna analyses*		Pfizer-BioNTech analyses^†^	
	SARS-CoV-2 test result		SARS-CoV-2 test result	
	Positive (case-patients) (n = 9,807)	Negative (control-patients) (n = 27,203)	Positive (case-patients) (n = 6,517)	Negative (control-patients) (n = 17,577)
**Age group, yrs**				
3	2,609 (27)	7,052 (26)	2,636 (40)	7,022 (40)
4	3,837 (39)	10,449 (38)	3,881 (60)	10,555 (60)
5	3,361 (34)	9,702 (36)	NA	NA
**Gender**				
Female	4,950 (50)	13,863 (51)	3,260 (50)	8,914 (51)
Male	4,817 (49)	13,162 (48)	3,230 (50)	8,550 (49)
Other	40 (0.4)	178 (1)	27 (0.4)	113 (1)
**Race and ethnicity** ^§^				
Black or African American, NH	2,075 (21)	5,167 (19)	1,352 (21)	3,373 (19)
Hispanic or Latino	2,960 (30)	7,140 (26)	1,965 (30)	4,492 (26)
White, NH	2,519 (26)	8,414 (31)	1,605 (25)	5,411 (31)
Other, NH	1,526 (16)	4,239 (16)	1,107 (17)	2,842 (16)
Unknown	727 (7)	2,243 (8)	488 (7)	1,459 (8)
**HHS testing site region** ^¶^				
Region 1	393 (4)	1,703 (6)	266 (4)	1,173 (7)
Region 2	631 (6)	2,360 (9)	458 (7)	1,606 (9)
Region 3	685 (7)	2,106 (8)	473 (7)	1,439 (8)
Region 4	2,718 (28)	7,469 (27)	1,753 (27)	4,654 (26)
Region 5	1,369 (14)	4,264 (16)	921 (14)	2,735 (16)
Region 6	2,084 (21)	4,297 (16)	1,343 (21)	2,774 (16)
Region 7	225 (2)	621 (2)	141 (2)	422 (2)
Region 8	118 (1)	316 (1)	77 (1)	203 (1)
Region 9	1,489 (15)	3,678 (14)	1,023 (16)	2,325 (13)
Region 10	95 (1)	389 (1)	62 (1)	246 (1)
SVI, mean (SD)**	0.6 (0.3)	0.5 (0.3)	0.6 (0.3)	0.5 (0.3)
**Period**				
Jul 4–Jul 31, 2022	3,885 (40)	6,330 (23)	2,626 (40)	4,253 (24)
Aug 1–Sep 18, 2022	3,839 (39)	9,588 (35)	2,501 (38)	6,198 (35)
Sep 19–Nov 30, 2022	1,083 (11)	7,500 (28)	765 (12)	4,805 (27)
Dec 1, 2022–Feb 5, 2023	1,000 (10)	3,785 (14)	625 (10)	2,321 (13)
**Caregiver-reported history of SARS-CoV-2 positive test result for child**				
None	8,662 (88)	21,120 (78)	5,788 (89)	13,794 (78)
Positive >90 days before current test	1,145 (12)	6,083 (22)	729 (11)	3,783 (22)
**SARS-CoV-2 test type**				
Rapid NAAT^††^	3,430 (35)	9,583 (35)	2,119 (33)	5,877 (33)
Laboratory-based NAAT^§§^	6,377 (65)	17,620 (65)	4,398 (67)	11,700 (67)
**Caregiver-reported one or more chronic underlying conditions for child** ^¶¶^				
No	9,551 (97)	26,501 (97)	6,354 (97)	17,139 (98)
Yes	256 (3)	702 (3)	163 (3)	438 (2)
Vaccination status***				
Unvaccinated	9,523 (97)	25,459 (94)	6,212 (95)	16,111 (92)
1 dose Moderna	107 (1)	402 (1)	NA	NA
2 doses Moderna	177 (2)	1,342 (5)	NA	NA
1 dose Pfizer-BioNTech	NA	NA	114 (2)	329 (2)
2 doses Pfizer-BioNTech	NA	NA	137 (2)	796 (5)
3 doses Pfizer-BioNTech	NA	NA	54 (1)	341 (2)

**Abbreviations:** HHS = U.S. Department of Health and Human Services; ICATT = Increasing Community Access to Testing; NA = not applicable; NAAT = nucleic acid amplification test; NH = non-Hispanic; SVI = Social Vulnerability Index; VE = vaccine effectiveness.

* Children who received Pfizer-BioNTech COVID-19 vaccine were excluded from the Moderna VE analyses.

^†^ Children who received Moderna COVID-19 vaccine were excluded from the Pfizer-BioNTech VE analyses.

^§^ Children whose caregiver reported NH ethnicity and any of the following for race were classified as Other, NH: American Indian or Alaska Native, Asian, Native Hawaiian or other Pacific Islander, or other race, or whose caregiver reported not Hispanic or Latino with no corresponding race chosen. Children whose caregiver did not report race and ethnicity were classified as unknown.

^¶^ Regions are defined by HHS and include only states, territories, and freely associated states with ICATT sites. U.S. Virgin Islands (Region 2) and Federated States of Micronesia, Guam, Marshall Islands, Northern Mariana Islands, Palau, and American Samoa (Region 9) were not included because they did not have pharmacies participating in ICATT. https://www.hhs.gov/about/agencies/iea/regional-offices/index.html

** SVI is a tool that uses U.S. Census Bureau data on 16 social factors to rank vulnerability by U.S. Census Bureau tract. The scale is from 0 to 1; higher SVIs represent more vulnerable communities. Tests with missing SVI data (<1% of total) were excluded from all analyses. Data in this study use 2020 SVI. https://www.atsdr.cdc.gov/placeandhealth/svi/data_documentation_download.html

^††^ Rapid NAAT was performed on-site on self-collected nasal swabs using ID Now (Abbott Diagnostics Scarborough, Inc.) and Accula (Thermo Fisher Scientific).

^§§^ Laboratory-based NAAT was performed on self-collected nasal swabs at contracted laboratories using a variety of testing platforms.

^¶¶^ Underlying conditions included on the questionnaire were heart conditions, high blood pressure, overweight or obesity, diabetes, current or former smoker, kidney failure or end stage renal disease, cirrhosis of the liver, and chronic lung disease (e.g., chronic obstructive pulmonary disease, moderate to severe asthma, cystic fibrosis, or pulmonary embolism). The questionnaire also included immunocompromising conditions; examples provided include immunocompromising medications, solid organ or blood stem cell transplant, HIV, or other immunocompromising conditions. Tests from children were excluded if the caregiver reported an immunocompromising condition.

*** Vaccination status categories are mutually exclusive. Percentages reflect column percentages among analytic sample and because exclusion criteria do not reflect vaccine coverage in the population of children seeking testing within ICATT.

**TABLE 2:** Monovalent vaccine effectiveness against symptomatic SARS-CoV-2 infection among young children, by vaccine product, number of doses, and time since last dose — Increasing Community Access to Testing program, United States, July 2022–February 2023

Vaccine product, age group, analysis period,* no. of doses (time since last dose)^†,§^	No. (%) of positive test results	No. (%) of negative test results	VE^¶^ (95% CI)
**Monovalent Moderna COVID-19 vaccine, children aged 3–5 yrs**			
**1-dose VE analysis, Jul 4, 2022–Feb 5, 2023**			
Unvaccinated (Ref)	9,523 (27)	25,459 (73)	Ref
1 dose only (2 wks–1 mo)	107 (21)	402 (79)	40 (26 to 52)
**2-dose VE analysis, Aug 1, 2022–Feb 5, 2023**			
Unvaccinated (Ref)	5,690 (23)	19,359 (77)	Ref
2 doses			
2 doses (2 wks–2 mos)	81 (10)	735 (90)	60 (49 to 68)
2 doses (3–4 mos)	58 (12)	437 (88)	36 (15 to 52)
2 doses (5–6 mos)**	NA	NA	NA
**Monovalent Pfizer-BioNTech COVID-19 vaccine, children aged 3–4 yrs**			
**1-dose VE analysis, Jul 4, 2022–Feb 5, 2023**			
Unvaccinated (Ref)	6,212 (28)	16,111 (72)	Ref
1 dose only (2 wks–1 mo)	114 (26)	329 (74)	19 (−1 to 35)
**2-dose VE analysis, Jul 25, 2022–Feb 5, 2023**			
Unvaccinated (Ref)	4,298 (25)	13,136 (75)	Ref
2 doses only (2 wks–3 mos)	137 (15)	796 (85)	40 (28 to 50)
**3-dose VE analysis, Sep 19, 2022–Feb 5, 2023** ^††^			
Unvaccinated (Ref)	1,273 (17)	6,275 (83)	Ref
3 doses only (2 wks–4 mos)	53 (13)	342 (87)	31 (7 to 49)

**Abbreviations:** NA = not applicable; Ref = referent group; VE = vaccine effectiveness.

* Different analysis periods were used for each vaccine product and dose number. Children became eligible to be included in each analysis 2 weeks after the initial date a child could have received each vaccine product and dose combination: 1 dose of Moderna and Pfizer-BioNTech on July 4, 2022; 2 doses of Pfizer-BioNTech on July 25, 2022; 2 doses of Moderna on August 1, 2022; and 3 doses of Pfizer-BioNTech on September 19, 2022.

^†^ Only month and year of receipt of each vaccine dose were reported from some participating pharmacies; therefore, the number of months between a vaccine dose and testing is a whole number calculated as the difference between the month and year of testing and the month and year of the vaccine dose. Tests from children for whom receipt of a third COVID-19 vaccine dose on or after December 2022 (when bivalent COVID-19 vaccine doses were recommended for this age group) was reported were excluded.

^§ ^For doses received in the same month or the month before SARS-CoV-2 testing, an additional question was asked to ascertain whether the dose was received ≥2 weeks before testing if the most recent vaccination date included only month and year. Only doses received ≥2 weeks before testing were included.

^¶^ VE = (1 − adjusted odds ratio) x 100. Odds ratios were calculated using multivariable logistic regression, adjusting for single year of age, gender, race, ethnicity, Social Vulnerability Index of the testing location, underlying conditions (presence versus absence), U.S. Department of Health and Human Services region of testing site, pharmacy chain conducting the test, local incidence (cases per 100,000 population by site county in the 7 days before test date), and testing calendar date.

** Moderna 2 dose VE at 5–6 months after receipt of the second dose did not meet precision threshold as CI width >50 percentage points, and thus data are not shown. Among second dose recipients, 165 and 43 children received a second dose of Moderna vaccine 5 months and 6 months before testing, respectively.

^††^ Pfizer-BioNTech 3-dose VE estimates did not have sufficient power to stratify by time since vaccination.

## Discussion

Postauthorization estimates of COVID-19 VE against symptomatic infection in young children indicate that complete primary series vaccination with either monovalent Moderna or Pfizer-BioNTech provides protection for children aged 3–5 and 3–4 years, respectively, against symptomatic infection for at least the first 4 months after vaccination. The goal of the U.S. COVID-19 vaccination program is to prevent severe disease and hospitalization (*9*); however, postauthorization VE against symptomatic infection provides important insight into vaccine protection, as estimates of VE against severe disease in this age group are not yet available. Effectiveness of mRNA vaccines has generally been higher against more severe outcomes than for symptomatic infection (*10*). Vaccination is an important tool for protecting children from COVID-19. Children should stay up to date with recommended COVID-19 vaccines, including completing the primary series; those who are eligible should receive a bivalent vaccine dose.

In this analysis, 1 dose of monovalent Moderna vaccine provided detectable protection against symptomatic infection in children aged 3–5 years; however, the point estimate only reflects the short period from 2 weeks after dose 1 to receipt of dose 2 (in the 2 weeks–1 month after the dose). Significant protection was not observed in the 2 weeks–1 month period after a single monovalent Pfizer-BioNTech vaccine dose. However, 2 Pfizer-BioNTech doses (which is an incomplete primary series for this age group) provided detectable protection against symptomatic infection, indicating that children who are awaiting their third dose had protection against symptomatic infection, but this VE is only reflective of protection provided during the interval between dose 2 and 3. In the Pfizer-BioNTech clinical trial, the prespecified immunobridging criteria were met after dose 3 but not after dose 2 among children aged 2–5 years (*3*). Receipt of a complete COVID-19 vaccination primary series is important to optimize vaccine-conferred protection in young children (*1*,*6*).

Several studies have demonstrated that monovalent mRNA VE wanes among older children and adults, particularly during Omicron variant predominance (*4*,*5*). The current analysis suggests that waning of complete monovalent Moderna primary series VE against symptomatic infection might occur among children aged 3–5 years by 3–4 months after the second dose based on point estimates (although CIs overlapped), similar to patterns seen in older children and adults in the first months after vaccination. Waning of monovalent Pfizer-BioNTech VE could not be assessed but is also likely based on analyses in older children and adults (*4*,*5*). Bivalent vaccines were introduced to address reduced VE against Omicron variants and waning protection (*9*). As of December 9, 2022, children aged 6 months–4 years receiving a Pfizer-BioNTech primary series are recommended to receive a monovalent vaccine for doses 1 and 2 and a bivalent vaccine as dose 3, and children aged 6 months–5 years who received the 2-dose Moderna primary series are recommended to receive a bivalent booster dose ≥2 months after completion of the primary series (*6*). Bivalent vaccines provide additional protection against infection and hospitalization in adults who have previously received monovalent COVID-19 vaccines (*7*,*8*); benefits in children are expected to be similar.

The findings in this report are subject to at least seven limitations. First, VE estimates for Moderna and Pfizer-BioNTech are not directly comparable because of different dates of eligibility for completion of the primary series, which might affect product-specific VE estimates. Decreased SARS-CoV-2 circulation during September 19, 2022–February 5, 2023 (when VE for a complete primary series for both products could be assessed), compared with that during August 1–September 18, 2022^†††††^ (when only Moderna complete primary series VE could be assessed) limited statistical power to estimate potential waning of 3-dose Pfizer-BioNTech VE. Second, vaccination coverage in this analysis is low, albeit higher than among children aged 2–4 years in the United States overall.^§§§§§^ Vaccinated children might be systematically different from unvaccinated children in COVID-19 risk or likelihood of seeking SARS-CoV-2 testing, which could bias VE results; thus, these early VE estimates should be considered preliminary. Third, data on hospitalization or severe outcomes are not available in ICATT. Fourth, vaccination status was reported by caregivers and was not verified, which could have resulted in misclassification of vaccination history. Fifth, this analysis reflects VE in children with a high prevalence of previous infection. By November–December 2022, 90% of U.S. children aged 6 months–4 years had evidence of infection-induced SARS-CoV-2 immunity^¶¶¶¶¶^; however, caregivers reported previous SARS-CoV-2 infection >3 months earlier for only approximately 20% of children in this analysis, and, therefore, the analysis was not adjusted for previous infection. Consequently, vaccine effectiveness in this analysis reflects the current situation among young children in the United States. Sixth, data were not collected on behaviors affecting COVID-19 risk (e.g., child care attendance), which could result in residual confounding. Finally, these VE estimates reflect circulation of a mix of Omicron sublineages.******

Complete monovalent Moderna and Pfizer-BioNTech primary series vaccination provided protection against symptomatic infection in children aged 3–5 and 3–4 years, respectively, for at least the first 4 months after vaccination. CDC will continue to monitor VE in young children. All children should stay up to date with recommended COVID-19 vaccines, including completing the primary series; those who are eligible should receive a bivalent vaccine dose.

**Summary**

**What is already known about this topic?**

Since June 2022, COVID-19 primary series vaccination has been recommended for young children with either Moderna for children aged 6 months–5 years or Pfizer-BioNTech for children aged 6 months–4 years; however, postauthorization vaccine effectiveness data are limited.

**What is added by this report?**

Complete monovalent Moderna and Pfizer-BioNTech primary series vaccination provides protection for children aged 3–5 and 3–4 years, respectively, against symptomatic SARS-CoV-2 infection for at least the first 4 months after vaccination.

**What are the implications for public health practice?**

Children should stay up to date with COVID-19 vaccines, including completing the primary series; those who are eligible should receive a bivalent vaccine dose. Continued vaccine effectiveness monitoring in young children is needed.

## Footnotes

* Moderna COVID-19 vaccine primary series consists of two 25 *μ*g doses separated by at least 4–8 weeks.

† Pfizer-BioNTech COVID-19 vaccine primary series consists of three 3 *μ*g doses, with at least 3–8 weeks between doses 1 and 2 and ≥8 weeks between doses 2 and 3.

§ At nearly all ICATT sites, test eligibility is restricted to persons aged ≥3 years. Therefore, this analysis was limited to children aged ≥3 years.

** https://www.cdc.gov/icatt/index.html (Accessed February 10, 2023).

†† During the analytic period, different versions of the questionnaire were used. Persons who reported a history of previous SARS-CoV-2 infection were also asked to report when the previous positive test result occurred and to select all applicable options. One version asked if the previous SARS-CoV-2 positive test result was within 90 days or ≥90 days ago, and another version asked if the previous positive test result was within the last week, between 1 week and 3 months ago, or >3 months ago.

§§ The following underlying conditions were included on the questionnaire: heart conditions, high blood pressure, overweight or obesity, diabetes, current or former smoker, kidney failure or end stage renal disease, cirrhosis of the liver, chronic lung disease (e.g., chronic obstructive pulmonary disease, moderate to severe asthma, cystic fibrosis, or pulmonary embolism), and immunocompromising conditions. For immunocompromising conditions, the following examples were provided on the questionnaire: immunocompromising medications, solid organ or blood stem cell transplant, HIV, or other immunocompromising conditions.

¶¶ Only month and year of receipt were reported for the vaccine dose from some participating pharmacies, and some questionnaires included the month and year of each dose, and others included the month and year of only the most recent dose. Therefore, the number of months between the most recent vaccine dose received and testing is a whole number calculated as the difference between the month and year of testing and the month and year of the most recent dose.

*** Children who received both Moderna and Pfizer-BioNTech COVID-19 vaccines were considered to have a mixed product regimen.

††† For doses received in the same month as or the month preceding SARS-CoV-2 testing, respondents were asked to specify whether the dose was received ≥2 weeks before testing if the most recent vaccination date was not directly reported. Only doses received ≥2 weeks before testing were included.

§§§ On December 9, 2022, children aged 6 months–5 years who had received 2 monovalent Moderna vaccine doses were recommended to receive a single bivalent booster dose ≥2 months after their last dose, and children aged 6 months–4 years who had received 2 monovalent Pfizer-BioNTech vaccine doses but had not yet received the third dose of the primary series were recommended to receive a bivalent vaccine dose as their third dose. In the analysis of Moderna VE, 38 tests were excluded from children who received a presumed bivalent third vaccine dose in December 2022 or later. In the analysis of Pfizer-BioNTech VE, 11 tests were excluded from children who received a presumed bivalent third vaccine dose in December 2022 or later.

¶¶¶ Children who received Pfizer-BioNTech COVID-19 vaccine were excluded from the analyses of Moderna VE, and children who received Moderna COVID-19 vaccine were excluded from the analyses of Pfizer-BioNTech VE.

**** Odds ratios were calculated using multivariable logistic regression, adjusting for single year of age, gender, race, ethnicity, Social Vulnerability Index of the testing location, underlying conditions (presence versus absence), U.S. Department of Health and Human Services region, pharmacy chain conducting the test, local incidence (cases per 100,000 population by site county in the 7 days before test date), and testing calendar date.

†††† Eligibility for each product and dose number combination began on the following dates: 1 dose of Moderna and Pfizer-BioNTech on July 4, 2022; 2 doses of Pfizer-BioNTech on July 25, 2022; 2 doses of Moderna on August 1, 2022; and 3 doses of Pfizer-BioNTech on September 19, 2022.

§§§§ VE for 1 dose was assessed at 2 weeks–1 month after the dose (i.e., tests conducted in the same month or month following the dose) to correspond to the recommended interval between doses 1 and 2 of at least 3–8 weeks for Pfizer-BioNTech and at least 4–8 weeks for Moderna. VE for 2 Pfizer-BioNTech doses was assessed at 2 weeks–3 months after dose 2 to correspond to the recommended interval of ≥8 weeks between doses 2 and 3.

¶¶¶¶ 45 C.F.R. part 46.102(l)(2), 21 C.F.R. part 56; 42 U.S.C. Sect. 241(d); 5 U.S.C. Sect. 552a; 44 U.S.C. Sect. 3501 et seq.

***** Among children with one or more COVID-19–like illness symptom with a negative SARS-CoV-2 NAAT in ICATT during July 4– February 5, 2023, before applying exclusion criteria, 20%, 19%, and 28% of those aged 3, 4, and 5 years had received ≥1 COVID-19 vaccine dose, respectively.

§§§§§ https://covid.cdc.gov/covid-data-tracker/#vaccination-demographics-trends (Accessed February 10, 2023).

¶¶¶¶¶ https://covid.cdc.gov/covid-data-tracker/#pediatric-seroprevalence (Accessed February 10, 2023).

****** https://covid.cdc.gov/covid-data-tracker/#variant-proportions (Accessed February 10, 2023).
