# *Erratum:* Vol. 72, No. 7

**DOI:** 10.15585/mmwr.mm7210a6

**Published:** 2023-03-10

In the report “Preliminary Estimates of Effectiveness of Monovalent mRNA Vaccines in Preventing Symptomatic SARS-CoV-2 Infection Among Children Aged 3–5 Years — Increasing Community Access to Testing Program, United States, July 2022–February 2023,” on page 181, the fifth sentence should have read, “By November–December 2022, **87%** of U.S. children aged 6 months–4 years had evidence of infection induced SARS-CoV-2 immunity^¶¶¶¶¶^; however, caregivers reported previous SARS-CoV-2 infection >3 months earlier for only approximately 20% of children in this analysis, and, therefore, the analysis was not adjusted for previous infection.”

